# Revisiting the Principles of Preservation in an Era of Pandemic Obesity

**DOI:** 10.3389/fimmu.2022.830992

**Published:** 2022-03-25

**Authors:** John T. Langford, Jenna R. DiRito, Natty Doilicho, Graylen R. Chickering, David A. Stern, Xinshou Ouyang, Wajahat Mehal, Gregory T. Tietjen

**Affiliations:** ^1^ Department of Surgery, Yale University School of Medicine, New Haven, CT, United States; ^2^ Department of Biomedical Engineering, Yale University, New Haven, CT, United States; ^3^ Section of Digestive Diseases, Yale University, New Haven, CT, United States

**Keywords:** obesity, cold storage, fasting, hibernation, metabolism

## Abstract

The current obesity epidemic has caused a significant decline in the health of our donor population. Organs from obese deceased donors are more prone to ischemia reperfusion injury resulting from organ preservation. As a consequence, these donors are more likely to be discarded under the assumption that nothing can be done to make them viable for transplant. Our current methods of organ preservation—which remain relatively unchanged over the last ~40 years—were originally adopted in the context of a much healthier donor population. But methods that are suitable for healthier deceased donors are likely not optimal for organs from obese donors. Naturally occurring models of acute obesity and fasting in hibernating mammals demonstrate that obesity and resilience to cold preservation-like conditions are not mutually exclusive. Moreover, recent advances in our understanding of the metabolic dysfunction that underlies obesity suggest that it may be possible to improve the resilience of organs from obese deceased donors. In this mini-review, we explore how we might adapt our current practice of organ preservation to better suit the current reality of our deceased donor population.

## Introduction

**Figure 1:**
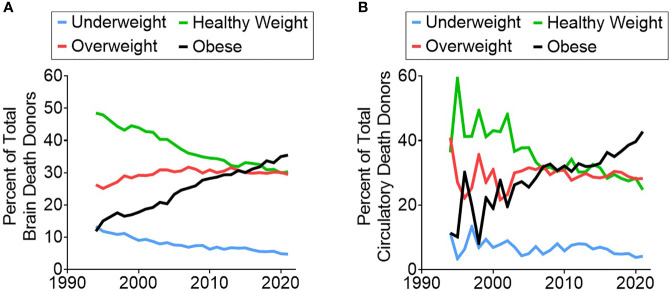
Trend showing the increasing rate of obese donors over time in both **(A)** brain death donors and **(B)** circulatory death donors.

Since Joseph Murray and David Hume performed the first successful transplant of a deceased donor organ in 1962, the rate of obesity in the U.S. has more than tripled from 12.8% to 42.4% (1, 2). In this same time span, abdominal solid organ transplantation has gone from a highly experimental technique to a reliable cure for end-stage organ failure; 1-year survival rates now exceed 90% for liver and 95% for kidney (3, 4). However, in current practice, these high success rates are dependent on selecting relatively healthy donor organs. Unfortunately, the continuing rise in obesity rates within our donor pool (**Figure 1**) often forces patients to choose between two undesirable options: accept the added risk that comes with an organ from a less healthy donor or risk dying on the waitlist before a better offer comes.

For recipients who choose to accept an organ from an obese donor, the added risk of post-transplant complications can be substantial (5, 6). In one study, livers from deceased donors with BMI >35 had a 156% increased likelihood of early allograft dysfunction (6). A recent retrospective analysis of 14 years of registry data—from 2000-2014—revealed significantly higher risk of death-censored graft failure in renal transplants when receiving an organ from a mildly obese donor, BMI 30-35, (HR = 1.10) or a very obese donor, BMI >35 (HR = 1.22) (5). This elevated risk was particularly pronounced in deceased donor organs suggesting that donor obesity sensitizes their organs to injury during cold-storage preservation. Given these adverse outcomes, it is not surprising that organs from obese donors are more likely to be discarded (7). However, the practice of throwing away all less-than-ideal organs is not a viable long-term strategy in our current era of pandemic obesity.

The current rationale for disproportionately discarding organs from obese donors is that obesity renders these organs more susceptible to ischemia reperfusion injury (IRI), thereby increasing the risk of complications to an unacceptable degree. This line of reasoning assumes that our current approaches to organ preservation—which remain relatively unchanged since they were initially developed ~40 years ago—are as optimal for organs from obese donors as they are for young healthy donors. In this Mini-Review, we propose an alternative perspective effective preservation of organs from obese donors will require new methods specifically tailored to the mechanisms of injury present in these organs. In support of our perspective we present protective adaptations in hibernating animals that allow these obese animals to survive cold storage like conditions. We further discuss how these adaptations relate to historic and contemporary studies on the benefits of fasting in animals and humans. We conclude by suggesting how these mechanistic insights might be translated to restorative interventions within the context of organ preservation.

## The Modern Western Diet and its Impact on Abdominal Organ Transplant

Nearly 50% of all adults in the U.S. have a chronic disease related to poor-quality diet (8). What constitutes a “healthy” diet should simply be based on scientific evidence. However, public recommendations can be complicated by multiple factors including politics, corporate interests, and complex scientific evidence that is difficult to interpret (9). While there is no common consensus on the best diet, there is agreement that both the total calories and macronutrient breakdown make a critical difference. Since 1970, Americans have increased their daily food intake by ~300 calories (10). Macronutrient breakdown has also changed substantially; our diets have become increasingly carbohydrate-based with a reduced percentage of calories from protein and fat. These changes to our diet have been linked to the concomitant rise of obesity and its associated pathologies (8).

The excess macronutrients in obese individuals leads to increased oxidative stress and can cause a predisposition to systemic inflammation and mitochondrial dysfunction (11–13). Abundant adipose tissue increases oxidative stress and the production of inflammatory cytokines (e.g. IL-6, TNF-alpha, MCP-1, and resistin) while decreasing anti-inflammatory mediators (e.g. adiponectin, Omentin, IL-10) (11, 14). Additionally, oxidative stress can also cause an increase in circulating free fatty acids and abnormal fat deposition in non-adipose tissues. This can result in further mitochondrial dysfunction and various forms of cell death such as lipotoxicity (15). Both effects can prime organs for damage during cold storage and reperfusion. For example, livers in cold storage from obese rats are shown to have increased rates of sinusoidal endothelial cell death compared to their lean counterparts (16). Inflammation can also prime transplanted organs for rejection by activating both the recipient immune system and the endothelium of the graft leading to an increase in the attachment and extravasation of T cells (17).

The metabolic and inflammatory dysfunction that occurs in organs from obese individuals are potentially reversible through lifestyle and diet changes. Many diets, such as the ketogenic diet or intermittent fasting, are suspected to not only promote weight loss but also repair the broken metabolic pathways caused by the average American diet (18). As one example, obese children put on a diet and lifestyle modification plan for three months had an increase in adiponectin and a decrease in inflammatory factors (C-reactive protein, IL-6) despite no significant weight loss (19). These findings suggest that it is possible to reduce or even reverse the underlying metabolic dysfunction and inflammation associated with the oxidative stress of obesity.

It is not ethically permissible to alter the diet of an organ donor with a prolonged intervention prior to organ retrieval. We therefore are restricted to evaluating if it is possible to target these pathologies during the preservation period that follows organ recovery. Or to state the question more succinctly: Can we better preserve obese organs to improve their resilience? To address this question, it is instructive to investigate existing examples from nature. Hibernating animals provide a unique model of a species that have evolved mechanisms to allow their organs to tolerate cold storage like conditions even when they are in an obese state.

## Hibernators as a Road Map Forward

Hibernating mammals demonstrate that obesity and resilience to cold hypoxia are not mutually exclusive. Some hibernating species (e.g., ground squirrels and marmots) are known to double their body mass prior to hibernation; adipose tissue can make up as much as 80% of body mass in these animals when they enter hibernation (20). Although this weight gain occurs in a shorter timeframe than most obese humans, their weight gain is still associated with insulin resistance, hyperinsulinemia, elevated triglyceride levels and buildup of fat stores (21). However, in contrast to obese organ donors, many hibernating animals have evolved physiologic adaptations that limit damage to their organs during hibernation. These adaptations—which include reductions in both immune activity and cell death—allow these animals to withstand levels of hypoxia and hypothermia that can be just as severe as cold-storage for deceased donor organs.

When animals, such as ground squirrels and bats, enter hibernation their organs are subjected to hypoxia and hypothermia. Nevertheless, they can withstand the IRI that occurs as they wake. The primary cell type involved in tissue destruction in IRI are neutrophils (22). It is therefore notable that hibernation triggers a stark ~90% reduction in the number of circulating leukocytes, with a significant decrease in mature neutrophils (23, 24). While this leaves the animals more susceptible to infection, It is believed that this trade-off ensures their organs can better withstand the IRI that results from the repeating cycles of torpor and arousal that occur during hibernation (23). While the mechanisms that drive IRI are complex and multi-factorial, it appears that preventing neutrophil induced tissue destruction may play a pivotal role in allowing hibernators to avoid organ damage despite their obese state.

Down regulation of cell death pathways—in particular ferroptosis (an iron-dependent form of cell death)—has also been shown to be a key adaptation of hibernating animals for resilience to cold hypoxia. Renal cells of hibernating animals are less susceptible to ferroptosis when compared to the same cells derived from human or rat tissues. However, when non-hibernating cells were given ferrostatin, an inhibitor of ferroptosis, cell survival was similar to the hibernating cell lines (25). In the 1970s, when UW solution was first created, our understanding of cell death was limited, but there are now many described forms of regulated cell death (26). Some regulated cell death modes are known to be immune-stimulatory such as pyroptosis and immunogenic cell death (26). Developing therapeutic methods to regulated cell death pathways may be crucial to improving the resilience of organs from obese donors.

The preceding examples demonstrate that hibernators adapt to increase resilience prior to entering hibernation. The key question then is what serves as the trigger for these adaptations? Emerging evidence suggest that the switch from the overfed state to a fasting state that occurs in parallel with the beginning of hibernation facilitates this process (27). Hindle and colleagues found that most of the differences in the liver proteome that distinguished active vs hibernating animals was similar to the signatures observed in fed versus fasted animals. The switch to the fasted state that occurs with beginning of the hibernation period is believed to prime these animals with the capacity to withstand the stress of IRI and support the metabolic reactivation during period of arousal (27). Fasting has also been shown to make organs of non-hibernating animals more resilient to cold ischemic injury (28).

## Fasting as a Pathway Forward

In animal models of stroke and myocardial infarction, fasting has been shown to protect against damage associated with IRI (29, 30). The improvement in outcomes from these preliminary studies led to the evaluation of whether other organ systems could also gain new resilience against ischemia from fasting. Mitchell et al. evaluated warm IRI in both kidney and liver in fasted and fed mice (31). In kidney models, mice that were fasted showed improved survival outcomes and decreased levels of acute tubular necrosis, serum urea and serum creatine. Similar results were observed in liver models where fasted mice had lower levels of ALAT and hemorrhagic necrosis (31). The benefits of fasting have also been demonstrated in animal transplant models (28, 32, 33).

The benefits of fasting in transplant were incidentally discovered by Southard and Belzer in 1993 (28). They initially thought fasting would mimic conditions of donors with extended ICU stays prior to donation and lead to adverse outcomes. However, rats fasted for 4 days had markedly improved survival compared to fed rats after both warm and cold ischemia insults (28). After 60 minutes of warm ischemia none (0/8) of the rats that received livers from fed donor survived, while 89% (8/9) of the rats that received livers from fasted rats survived. After 44 hours of cold ischemia, only 29% (2/7) of rats that received livers from fed rats survived, while 83% (5/6) of rats that received livers from fasted rats survived. These impressive survival benefits have been confirmed in additional follow up studies (32, 33). While the benefits of fasting have been shown in animal models, fasting is not a feasible intervention in human organ donors. Therefore, if we wish to modulate these pathways to improve preservation of organs from obese donors, we need to understand what pathways to modulate pharmacologically that will enable us to target these pathways prior to or during cold storage.

Studies to understand the potential mechanisms for the survival benefits of fasting have found similar mechanisms to hibernators: reductions in both inflammation as well as cell death. For example, fasted rats given intraperitoneal injections of zymosan—a glucan that produces sterile inflammation—had lower levels of TNF-α compared to fed controls (32). It is postulated that the decreased production of inflammatory factors from donors leads to a lesser immune response in recipients and as a result, less injury. Sun et al. demonstrated that fasting also decreases apoptosis in rat donor livers. Apoptosis was the same at 24 hours of cold storage in both fasted and fed rats, but after 6 hours of reperfusion there were significantly more dead sinusoidal endothelial cells in the fed group than in the fasted group (33). The difference in post reperfusion cell death demonstrates that there may be underlying metabolic changes that make fasted tissues more resilient to IRI. This suggests that intervening on these pathways could provide an avenue to improving obese donor organs’ resilience to the stresses of cold storage. This is one of many strategies that may work to improve the current practice of cold preservation.

## Improving Current Cold Storage Techniques

It appears fasting in both hibernating and non-hibernating animals reduces inflammation and limits cell death during reperfusion injury. These are the exact vulnerabilities that obesity appears to exacerbate. The stresses of cold storage can potentially be mitigated by adding therapeutic these specific to target these specific pathways in cold storage solutions or altering the storage conditions themselves.

Ketone bodies, which are a naturally produced from breakdown of fatty acids in a fasted state. They not only function as a fuel source for cells, but also act as signaling molecules (18). In particular, β-hydroxybutyrate, which is one of three endogenously produced ketones, has been shown to be protective against ischemia-reperfusion injury in a mouse model *in vivo* and human cells *in vitro* (34). TUNEL positive cells were decreased by inducing endogenous production of β-hydroxybutyrate *via* fasting or by administration of exogenous β-hydroxybutyrate. This effect is believed to be mediated through anti-pyroptotic effects by inducing FOXO3, an up-stream transcription factor for pyroptosis.

One strategy that may be of interest is intervention in the peroxisome proliferator-activated receptor (PPAR) pathway. Obesity is associated with the development of metabolic syndrome which is a cluster of conditions including increased fat around the waist, elevated cholesterol, elevated triglyceride and increased risk of heart disease and type 2 diabetes. PPAR agonists are medications which are currently used to treat elevated triglycerides or blood glucose but have been shown to be effective in managing metabolic syndrome (35). Interestingly, PPARs are upregulated in fasting and play an important role in metabolic regulation (36). Not only are PPARs directly involved in metabolic syndrome and fasting but activation of PPARα has been shown to play a critical role in decreasing apoptosis and inflammation during renal IRI in a mouse model (37). Pre-treatment of mice with a PPARα activator, docosahexaenoic acid, significantly decreased the apoptotic and inflammatory responses compared to untreated wild type mice while PPARα knockout mice had increased apoptosis and inflammation (37).

Another important pathway to consider is the NLRP3 Inflammasome. The NLRP3 inflammasome is a multi-protein complex consisting of NLRP3 (sensor), ASC (adaptor), and Caspase-1 (effector). It is activated by a wide range of stimulus and is associated with sterile inflammation in obesity (38). It then causes the release of IL-1β, IL-18 and leads to pyroptotic cell death (39). In particular, NLRP3 has been shown to be important in ischemia reperfusion of the brain and heart in animal models (40). Using a model of *ex-vivo* ischemia reperfusion demonstrated that using INF4E, a NLRP3 inflammasome inhibitor, reduced infarct size, lactate dehydrogenase release and improved left ventricular pressure. Interestingly, fasting decreases NLRP3 inflammasome in humans and is believed to be due to SIRT3- mediated activation of superoxide dismutase 2 (41). By targeting the obesity-induced inflammation and its consequences we could potentially target several pathways to improve outcomes from obese donor organs.

## Conclusions

**Figure 2:**
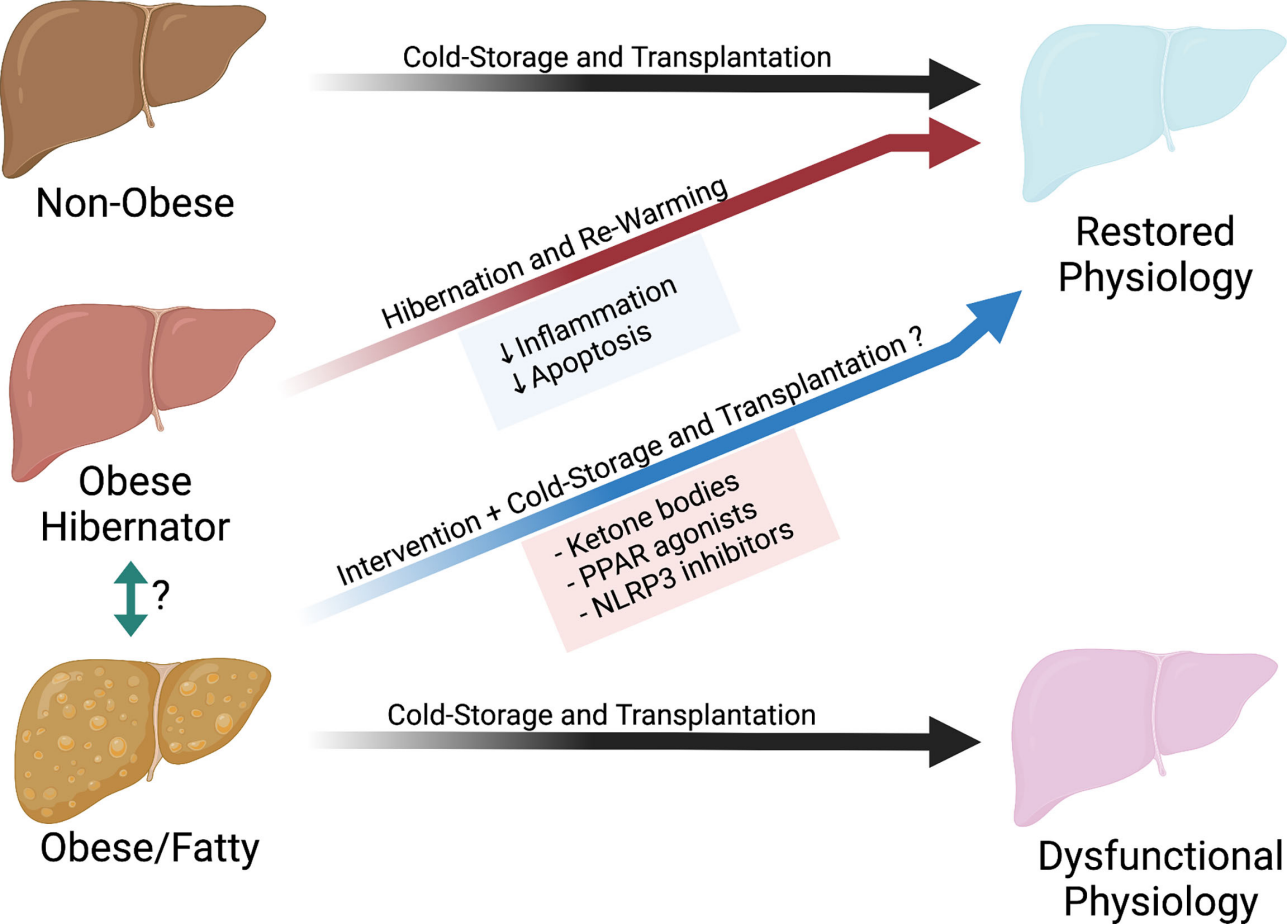
Potential pathway to restore organs from obese donors.

Our understanding and applied practices of cold storage has been based on ideal organs from a healthy population. However, as obesity rates increase in the population, it is of paramount importance to understand the effects of cold storage on obese organs (**Figure 2**). We can build on the previous work of Belzer, Southard and others by incorporating different fields into transplant science, such as hibernating animal models or the nutritional science of fasting. This will allow us to expand our understanding on the modes of failure during cold storage. We can then implement new therapeutics and new technologies such as machine perfusion to treat and rehabilitate these organs. This has the potential to both improve outcomes and increase the number of transplantable organs for patients on the waitlist.

## Author Contributions

Concept and Design: JL and GT. Writing: JL. Critical Revisions: JD, ND, GC, DS, XO, WM, and GT. All authors contributed to the article and approved the submitted version.

## Funding

Supported by the National Institutes of Health Grant T32GM086287 and R01DK124420.

## Conflict of Interest

GT is the co-founder of Revalia Bio.

The remaining authors declare that the research was conducted in the absence of any commercial or financial relationships that could be construed as a potential conflict of interest.

## Publisher’s Note

All claims expressed in this article are solely those of the authors and do not necessarily represent those of their affiliated organizations, or those of the publisher, the editors and the reviewers. Any product that may be evaluated in this article, or claim that may be made by its manufacturer, is not guaranteed or endorsed by the publisher.

## References

[1] Hales C Carroll M Fryar C Ogden C. Prevalence of Obesity and Severe Obesity Among Adults: United States, 2017–2018. Hyattsville, MD: National Center for Health Statistics (2020).

[2] Flegal KM Carroll MD Kuczmarski RJ Johnson CL. Overweight and Obesity in the United States: Prevalence and Trends, 1960-1994. Int J Obes Relat Metab Disord (1998) 22(1):39–47. doi: 10.1038/sj.ijo.0800541 9481598

[3] Hart A Smith JM Skeans MA Gustafson SK Wilk AR Castro S. Optn/Srtr 2018 Annual Data Report: Kidney. Am J Transplant (2020) 20(s1):20–130. doi: 10.1111/ajt.15672 31898417

[4] Kwong A Kim WR Lake JR Smith JM Schladt DP Skeans MA. Optn/Srtr 2018 Annual Data Report: Liver. Am J Transplant (2020) 20 Suppl s1:193–299. doi: 10.1111/ajt.15674 31898413

[5] Naik AS Zhong Y Parasuraman R Doshi M Norman S Lu Y. The Temporal and Long-Term Impact of Donor Body Mass Index on Recipient Outcomes After Kidney Transplantation - a Retrospective Study. Transpl Int (2020) 33(1):59–67. doi: 10.1111/tri.13505 31478267

[6] Hudcova J Qasmi ST Ruthazer R Waqas A Haider SB Schumann R. Early Allograft Dysfunction Following Liver Transplant: Impact of Obesity, Diabetes, and Red Blood Cell Transfusion. Transplant Proc (2020) 53(1):199–23. doi: 10.1016/j.transproceed.2020.02.168 32690312

[7] Mohan S Chiles MC Patzer RE Pastan SO Husain SA Carpenter DJ. Factors Leading to the Discard of Deceased Donor Kidneys in the United States. Kidney Int (2018) 94(1):187–98. doi: 10.1016/j.kint.2018.02.016 PMC6015528 29735310

[8] Wilson MM Reedy J Krebs-Smith SM. American Diet Quality: Where it is, Where it is Heading, and What it Could be. J Acad Nutr Diet (2016) 116(2):302–10.e1. doi: 10.1016/j.jand.2015.09.020 PMC4733413 26612769

[9] Nestle M. Food Politics: How the Food Industry Influences Nutrition and Health. Rev. And Expanded Ed. California Studies in Food and Culture Vol. xviii. . Berkeley: University of California Press (2007). p. 486.

[10] Centers for Disease, C. and Prevention. Trends in Intake of Energy and Macronutrients–United States, 1971-2000. MMWR Morbidity Mortality Weekly Rep (2004) 53(4):80–2. 14762332

[11] Ellulu MS Patimah I Khaza’ai H Rahmat A Abed Y. Obesity and Inflammation: The Linking Mechanism and the Complications. Arch Med science: AMS (2017) 13(4):851–63. doi: 10.5114/aoms.2016.58928 PMC5507106 28721154

[12] Bournat JC Brown CW. Mitochondrial Dysfunction in Obesity. Curr Opin Endocrinol Diabetes Obes (2010) 17(5):446–52. doi: 10.1097/MED.0b013e32833c3026 PMC5001554 20585248

[13] Mosbah IB Rosello-Catafau J Alfany-Fernandez I Rimola A Parellada PP Mitjavila MT. Addition of Carvedilol to University Wisconsin Solution Improves Rat Steatotic and Nonsteatotic Liver Preservation. Liver Transpl (2010) 16(2):163–71. doi: 10.1002/lt.21968 20104484

[14] Makki K Froguel P Wolowczuk I. Adipose Tissue in Obesity-Related Inflammation and Insulin Resistance: Cells, Cytokines, and Chemokines. ISRN Inflammation (2013) p:139239. doi: 10.1155/2013/139239 PMC3881510 24455420

[15] Schrauwen P Schrauwen-Hinderling V Hoeks J Hesselink MKC. Mitochondrial Dysfunction and Lipotoxicity. Biochim Biophys Acta (2010) 1801(3):266–71. doi: 10.1016/j.bbalip.2009.09.011 19782153

[16] Fukumori T Ohkohchi N Tsukamoto S Satomi S. The Mechanism of Injury in a Steatotic Liver Graft During Cold Preservation. Transplantation (1999) 67(2):195–200. doi: 10.1097/00007890-199901270-00002 10075580

[17] Mori DN Kreisel D Fullerton JN Gilroy DW Goldstein DR. Inflammatory Triggers of Acute Rejection of Organ Allografts. Immunol Rev (2014) 258(1):132–44. doi: 10.1111/imr.12146 PMC3939032 24517430

[18] de Cabo R Mattson MP. Effects of Intermittent Fasting on Health, Aging, and Disease. New Engl J Med (2019) 381(26):2541–51. doi: 10.1056/NEJMra1905136 31881139

[19] Balagopal P George D Yarandi H Funanage V Bayne E. Reversal of Obesity-Related Hypoadiponectinemia by Lifestyle Intervention: A Controlled, Randomized Study in Obese Adolescents. J Clin Endocrinol Metab (2005) 90(11):6192–7. doi: 10.1210/jc.2004-2427 16131584

[20] Boyer BB Barnes BM. Molecular and Metabolic Aspects of Mammalian Hibernation: Expression of the Hibernation Phenotype Results From the Coordinated Regulation of Multiple Physiological and Molecular Events During Preparation for and Entry Into Torpor. BioScience (1999) 49(9):713–24. doi: 10.2307/1313595

[21] Martin SL. Mammalian Hibernation: A Naturally Reversible Model for Insulin Resistance in Man? Diabetes Vasc Dis Res (2008) 5(2):76–81. doi: 10.3132/dvdr.2008.013 18537093

[22] Huang Y Rabb H Womer KL. Ischemia-Reperfusion and Immediate T Cell Responses. Cell Immunol (2007) 248(1):4–11. doi: 10.1016/j.cellimm.2007.03.009 17942086 PMC2211448

[23] Yasuma Y McCarron RM Spatz M Hallenbeck JM. Effects of Plasma From Hibernating Ground Squirrels on Monocyte-Endothelial Cell Adhesive Interactions. American Journal of Physiology-Regulatory. Integr Comp Physiol (1997) 273(6):R1861–9. doi: 10.1152/ajpregu.1997.273.6.R1861 9435638

[24] Bouma HR Carey HV Kroese FG. Hibernation: The Immune System at Rest? J Leukoc Biol (2010) 88(4):619–24. doi: 10.1189/jlb.0310174 20519639

[25] Hendriks KDW Joschko CP Hoogstra-Berends F Heegsma J Faber KN Henning RH. Hibernator-Derived Cells Show Superior Protection and Survival in Hypothermia Compared to non-Hibernator Cells. Int J Mol Sci (2020) 21(5). doi: 10.3390/ijms21051864 PMC7084219 32182837

[26] Galluzzi L Vitale I Aaronson SA Abrams JM Adam D Agostinis P. Molecular Mechanisms of Cell Death: Recommendations of the Nomenclature Committee on Cell Death 2018. Cell Death Differ (2018) 25(3):486–541. doi: 10.1038/s41418-018-0102-y 29362479 PMC5864239

[27] Hindle AG Grabek KR Epperson LE Karimpour-Fard A Martin SL. Metabolic Changes Associated With the Long Winter Fast Dominate the Liver Proteome in 13-Lined Ground Squirrels. Physiol Genomics (2014) 46(10):348–61. doi: 10.1152/physiolgenomics.00190.2013 PMC4042184 24642758

[28] Sumimoto R Southard JH Belzer FO. Livers From Fasted Rats Acquire Resistance to Warm and Cold Ischemia Injury. Transplantation (1993) 55(4):728–32. doi: 10.1097/00007890-199304000-00008 8475543

[29] Yu ZF Mattson MP. Dietary Restriction and 2-Deoxyglucose Administration Reduce Focal Ischemic Brain Damage and Improve Behavioral Outcome: Evidence for a Preconditioning Mechanism. J Neurosci Res (1999) 57(6):830–9. doi: 10.1002/(SICI)1097-4547(19990915)57:6<830::AID-JNR8>3.0.CO;2-2 10467254

[30] Chandrasekar B Nelson JF Colston JT Freeman GL. Calorie Restriction Attenuates Inflammatory Responses to Myocardial Ischemia-Reperfusion Injury. Am J Physiol Heart Circ Physiol (2001) 280(5):H2094–102. doi: 10.1152/ajpheart.2001.280.5.H2094 11299211

[31] Mitchell JR Verweij M Brand K Ven M Goemaere N Engel S. Short-Term Dietary Restriction and Fasting Precondition Against Ischemia Reperfusion Injury in Mice. Aging Cell (2010) 9(1):40–53. doi: 10.1111/j.1474-9726.2009.00532.x 19878145 PMC3412229

[32] Nishihara M Sumimoto R Sakimoto H Sanada O Fukuda Y Southard JH. TNF-Alpha and Heat-Shock Protein Gene Expression in Ischemic-Injured Liver From Fasted and non-Fasted Rats. Role of Donor Fasting in the Prevention of Reperfusion Injury Following Liver Transplantation. Transpl Int (1998) 11 Suppl 1:S417–20. doi: 10.1111/j.1432-2277.1998.tb01171.x 9665030

[33] Sun X Kimura T Kobayashi T Noriki S Imamura Y Fukuda M. Viability of Liver Grafts From Fasted Donor Rats: Relationship to Sinusoidal Endothelial Cell Apoptosis. J Hepatobiliary Pancreat Surg (2001) 8(3):268–73. doi: 10.1007/s005340170027 11455490

[34] Tajima T Yoshifuji A Matsui A Itoh T Uchiyama K Kanda T. Beta-Hydroxybutyrate Attenuates Renal Ischemia-Reperfusion Injury Through its Anti-Pyroptotic Effects. Kidney Int (2019) 95(5):1120–37. doi: 10.1016/j.kint.2018.11.034 30826015

[35] Botta M Audano M Sahebkar A Sirtori CR Mitro N Ruscica M. PPAR Agonists and Metabolic Syndrome: An Established Role? Int J Mol Sci (2018) 19(4). doi: 10.3390/ijms19041197 PMC5979533 29662003

[36] Ruan X Zheng F Guan Y. Ppars and the Kidney in Metabolic Syndrome. Am J Physiol Renal Physiol (2008) 294(5):F1032–47. doi: 10.1152/ajprenal.00152.2007 18234957

[37] Chen HH Chen TW Lin H. Prostacyclin-Induced Peroxisome Proliferator-Activated Receptor-Alpha Translocation Attenuates NF-Kappab and TNF-Alpha Activation After Renal Ischemia-Reperfusion Injury. Am J Physiol Renal Physiol (2009) 297(4):F1109–18. doi: 10.1152/ajprenal.00057.2009 19640904

[38] Traba J Kwartend-Siaw M Okoli TC Li J Huffstutler RD Bray A. Fasting and Refeeding Differentially Regulate NLRP3 Inflammasome Activation in Human Subjects. J Clin Invest (2015) 125(12):4592–600. doi: 10.1172/JCI83260 PMC4665779 26529255

[39] Swanson KV Deng M Ting JP. The NLRP3 Inflammasome: Molecular Activation and Regulation to Therapeutics. Nat Rev Immunol (2019) 19(8):477–89. doi: 10.1038/s41577-019-0165-0 PMC7807242 31036962

[40] Mastrocola R Penna C Tullio F Femmino S Nigro D Chiazza F. Pharmacological Inhibition of NLRP3 Inflammasome Attenuates Myocardial Ischemia/Reperfusion Injury by Activation of RISK and Mitochondrial Pathways. Oxid Med Cell Longev (2016) p:5271251. doi: 10.1155/2016/5271251 PMC5178375 28053692

[41] Traba J Geiger S Kwarteng-Siaw M Han K Hyuk Ra O Siegel R. Prolonged Fasting Suppresses Mitochondrial NLRP3 Inflammasome Assembly and Activation via SIRT3-Mediated Activation of Superoxide Dismutase 2. J Biol Chem (2017) 292(29):12153–64. doi: 10.1074/jbc.M117.791715 PMC5519366 28584055

